# Physiological and biochemical mechanisms of grain yield loss in fumitory (*Fumaria parviflora* Lam.) exposed to copper and drought stress

**DOI:** 10.1038/s41598-023-45103-5

**Published:** 2023-10-20

**Authors:** Mansoureh Tashakorizadeh, Pooran Golkar, Mohammad Reza Vahabi, Mansour Ghorbanpour

**Affiliations:** 1 Forests and Rangelands Research Department, Kerman Agricultural and Natural Resources Research and Education Center, Agricultural Research Education and Extension Organization (AREEO), Kerman, Iran; 2 Department of Natural Resources, Isfahan University of Technology, Isfahan, 8415683111 Iran; 3 Department of Medicinal Plants, Faculty of Agriculture and Natural Resources, Arak University, Arak, 38156-8-8349 Iran

**Keywords:** Physiology, Plant sciences

## Abstract

Soil contamination with heavy metals adversely affects plants growth, development and metabolism in many parts of the world including arid and semi-arid regions. The aim of this study was to investigate the single and combined effects of drought and copper (Cu) stresses on seed yield, and biochemical traits of *Fumaria parviflora* in a split – factorial experiment at Research Field of Payam-E-Noor university of Kerman during 2019. The collected seeds from two Cu contaminated regions were evaluated under drought and Cu (0, 50, 150, 300, and 400 mg/kg) stresses. Drought stress levels were depletion of 50% (D_1_), 70% (D_2_) and 85% (D_3_) soil available water. The individual effects of drought and copper stresses were similar to each other as both reduced seed yield. The highest seed yield was observed at Cu concentration of 50 mg/kg under non-drought stress conditions. The maximum values of malondialdehyde (0.47 µmol/g), proline (2.45 µmol/g FW), total phenolics (188.99 mg GAE/g DW) and total flavonoids (22.1 mg QE/g DW) were observed at 400 mg/kg Cu treatment. However, the strongest antioxidant activity (83.95%) through DPPH assay, and the highest total soluble carbohydrate (115.23 mg/g DW) content were observed at 300 and 150 mg/kg Cu concentration under severe drought stress, respectively. The highest amount of anthocyanin (2.18 µmol/g FW) was observed at 300 mg/kg Cu and moderate drought stress. The findings of this study showed a high tolerance of *F*. *parviflora* plant to moderate drought stress and Cu exposure up to 150 mg/kg by modulating defense mechanisms, where grain yield was slightly lower than that of control. The results could also provide a criterion for the selection of tolerance species like *F. parviflora* for better acclimatization under Cu mines and/or agricultural contaminated soils subjected to drought stress.

## Introduction

Different environmental biotic and abiotic stresses are the major factors affecting crop yields and productivity worldwide^1^. Abiotic stresses such as heavy metals affect various metabolic pathways and the growth performance of plants^2^. Moreover, drought stress is also known as an important concern which threatens the required food demand for the world’s growing population^3^. The intensity of drought stress is becoming more frequent due to decrease in rainfall and variations in climatic patterns^4^. Plants modulate the deleterious effects of drought stress by activating the antioxidant defense system which triggering a wide range of physio-chemical response^5–7^.

Heavy metals stresses effects on plants productivity which has widely been studied under naturally and industrially contaminated soils^8^. Copper (Cu) is considered as a micronutrient element among heavy metals, which is an essential element for plant cells function, metabolisms and their structure^9^. Furthermore, Cu act as an electron transporter, and regulates the cellular redox state^10^. Cu concentrations (at ˃ 20 mg/kg in medicinal plants according to the United States Food and Drug Administration (US-FDA) can become toxic. Excessive concentrations of Cu could have toxic effects in plants, which cause disruptions in growth and physiology^11,12^ through induction of oxidative stress and metabolic abnormalities^13^. A rapid change in industrialization, urbanization and production of industrial and agricultural waste products have increased the levels of different heavy metals in environment, which induce potential adverse health effects in humans and ecosystems^13,14^. Consequently, contaminations of soil with toxic levels of Cu enters in plant tissues and may lead to severe health problems in consumers^12^.

Multiple environmental stresses trigger a wide variety of plant responses ranging from biochemical, physiological and alterations at molecular levels^2,15^. The adaptability of plants to unfavorable conditions depends on the types of plant defense systems against stresses^4^. Different antioxidative systems in plants (enzymatic and non-enzymatic antioxidants) scavenge the reactive oxygen species (ROS) (such H_2_O_2_, O_2_^·−^ and OH^·^) generation under environmental stresses^16^. Different enzymatic antioxidants such as ascorbate peroxidase (APX), catalase (CAT), superoxide dismutase (SOD), glutathione peroxidase (GP) and glutathione reductase (GR) diminish the levels of H_2_O_2_ and lipid hydroperoxide, and thus they are important in the prevention of lipid peroxidation and maintaining the structure and function of plant cell membranes^17^. Non-enzymatic antioxidants such as glutathione, ascorbate, tocopherols, carotenoids, and flavonoids intercept and terminate free radical chain reactions, which have solubility in the water or lipids^17,18^. Increase in ROS generation under environmental stresses initiate different defensive mechanisms with an increase in the contents of secondary metabolites (SMs) such as phenolics, flavonoids and anthocyanins, which are ascribed vital role in plant antioxidant responses^16^. The other plant responses to environmental stresses includes the increase levels of osmolites like proline and soluble carbohydrates^19^.

*Fumaria parviflora* Lam. (Fumariaceae) considered as a valuable medicinal herb^20^. It is native to Europe, Asia, and Africa and has different vernacular names depending on the region. The herb possesses excellent medicinal properties such as diuretic, expectorant, stomach stimulant, blood purifier, appetizing, astringent in addition to its narcotic properties^21^. Under natural environmental conditions, plants might simultaneously be exposed to multiple types of abiotic stresses^22^. Therefore, the response of plants to combined stresses can not necessarily be extrapolated from their response to an individual stress^22^. Cultivation of medicinal plants is usually done under conditions with several types of environmental stresses, which results in reduced plant growth and yield^23^. Hence, it becomes important to evaluate the response of medicinal plants species exposed to combination of different environmental stresses.

The co-existence of drought and heavy metal stresses undesirably influence soil fertility, which hamper growth and development of plants^24,25^. Moreover, the combined effects of two stresses may trigger the incidence of the production of osmotic and oxidative constraints which negatively affect plant development and metabolic functions^24^. Although, a few studies have revealed the different ecological and physiological responses of plants to both water deficit and heavy metal stress^14,24,26^, no study was found on the combined effects of copper and drought stresses on medicinal plants. There is a deficiency of understanding of the effects of drought and Cu levels singly or in their combinations on different physiological and biochemical traits in medicinal plants. Therefore, the aims of the present study were to investigate the single and combined effects of Cu and drought stresses on seed yield and some biochemical features in *Fumaria parviflora* under field conditions. The findings of this study may help to add some new insights about how a suitable medicinal plant cope with detrimental effects of extreme drought and heavy metal toxicity in an area duly affected by these combined stresses.

## Results

The results of ANOVA showed highly significant (*p* < 0.01) effect of drought stress on all the studied traits (Table 1). The region had not significant effect on each of the traits, but there was a highly significant effect (*p* < 0.01) for zones on grain yield, proline, TSC, anthocyanins, TPC and TFD (Table 1). According to results of ANOVA, Cu had significant effect on all the studied traits (Table 1). The environment × drought interaction effects was significant (*p* < 0.01) only on TPC (Table 1). The interaction effects of environment × Cu was significant on grain yield, proline, TPC and TFD (*p* < 0.01; Table 1. Also the interaction effects of Cu × drought was significant on all the studied traits (*p* < 0.01; Table 1).

**Table 1:** Analysis of variance for the interaction effects of zones, Cu and drought stresses on *F. parviflora* under field condition.

Source of variation	Mean squares								
	DF	Grain yield	MDA	Proline	TSC	Anthocyanin	TPC	TFD	Antioxidant activity
Repetition	2	134.5**	0.39**	1.06**	422.17**	0.84**	3616.9**	6.99**	451.8**
Drought	2	18,931.9**	0.31**	8.41**	17,172.6**	9.25**	14,129.9**	1248.2*	1952.31**
Error (A)	4	0.57	0.002	0.01	1.45	0.0006	31.66	0.13	0.74
Environment (Env)	7	0.59**	0.03**	0.05	453.3*	0.16**	9955.9**	28.75*	112.11*
Region	1	0.08	0.09	0.12	2565.9	0.009	64,619.7	193.2	165.5
Zone	3	10.55**	0.03	0.08**	177.45**	0.35**	1684.7**	1.03**	179.92
Region × zone	3	2.47	0.001	0.04	14.88	0.019	5.84	1.65	25.15
Copper	4	4288.8**	0.04*	14.59**	30,837.9*	3.12**	32,849.1**	280.72**	14,307.6**
Env × drought	14	2.36	0.0005	0.004	7.38	0.005	237.52**	0.6	26.24
Copper × drought	8	137.66**	0.1*	1.22**	3491.66*	2.22**	1252.9**	24.12**	306.67*
Env × copper	28	9.11**	0.001	0.02*	57.79	0.07	520.16**	2.58**	34.21
Env × copper × drought	56	1.48**	0.0003	0.002*	3.74	0.002**	37.39**	0.1**	27.82
Error (B)	234	1.07	0.001	0.015	3.39	0.004	15.93	0.1	3.06

*MDA* malondialdehyde, *TSC* total soluble carbohydrate, *TPC* total phenolics content, *TFD* total flavonoids.

*and ** are significant at *p* < 0.05 and *p* < 0.01, respectively.

### Effects of zone on studied traits

**Figure 1:**
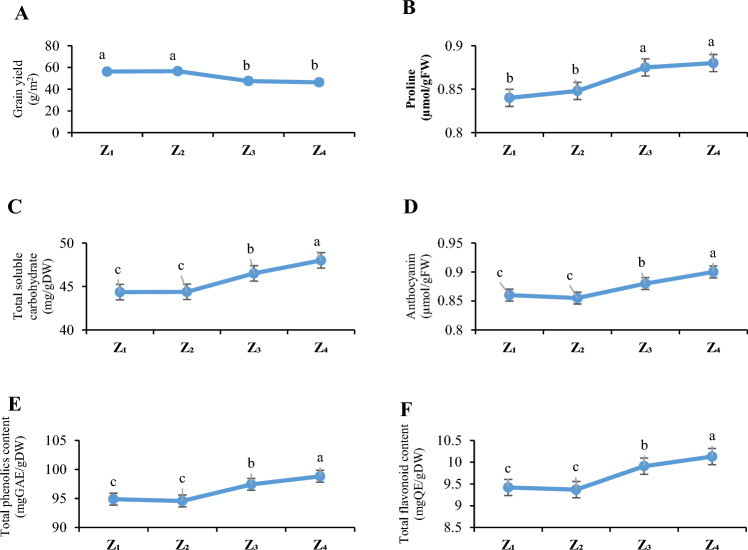
The effect of zones on grain yield (**A**), proline (**B**), Total soluble carbohydrates (**C**) anthocyanin (**D**), total phenolics content (**E**) and total flavonoids content (**F**) of *F. parviflora*. Different letters indicate significant differences (*p* < 0.05) between different zones (means ± SE). Z_1_: Zone 1. Z_2_: Zone 2. Z_3_: Zone 3. Z_4_: Zone 4.

The originated plants from Z_3_ and Z_4_ showed lower level of grain yield and higher levels of biochemical traits than Z_1_ and Z_2_ (Fig. 1). The grain yield decreased by an average of 115.43% in Z_3_ and Z_4_ compared to Z_1_ and Z_2_ (Fig. 1). The biochemical traits increased by an average of 116.44% for proline 115.63% for TSC, 119.34% for anthocyanin, 114.68% for TPC and 115.98% for TFD in Z_3_ and Z_4_ compared to Z_1_ and Z_2_ (Fig. 1).

### Effects of Cu, drought stress and Cu × drought interaction on studied traits

#### Grain yield

**Figure 2:**
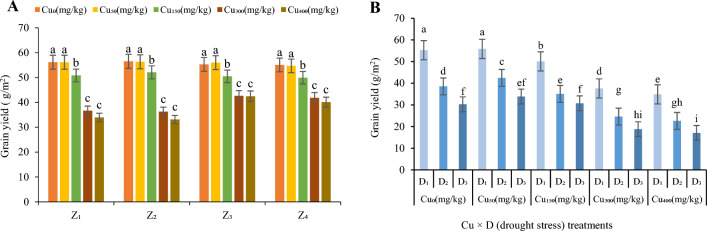
The effect of Cu stress on grain yield of *F. parviflora* under field conditions (**A**). The means for the interaction effects of Cu and drought stresses on grain yield (**B**). Different letters for each figure indicate significant differences at *p* < 0.05. Copper concentration (mg/kg). Seed originated zones: Z_1_: Zone 1. Z_2_: Zone 2. Z_3_: Zone 3. Z_4_: Zone 4. D_1_: Control, D_2_: moderate drought stress; D_3_: severe drought stress.

The highest grain yield was observed at Cu_0_ and Cu_50_ (56.16 g/m^2^ in Z_1_, 56.51 g/m^2^ in Z_2_, 55.98 g/m^2^ in Z_3_ and 54.67 g/m^2^ in Z_4_; Fig. 2) while the plants in the soil treated with Cu_400_ showed the lowest grain yield in all zones (33.94 g/m^2^ in Z_1_, 33.1 g/m^2^ in Z_2_, 36.45 g/m^2^ in Z_3_ and 36.08 g/m^2^ in Z_4_; Fig. 2A). The concentrations of Cu_0_ and Cu_50_ were appropriate for grain yield, but with increasing Cu concentration, a reduction in grain yield was observed, which was significant (*p* ≤ 0.01). This reduction in Cu_150_ (10%), was less than concentrations of Cu_300_ (40% in Z_1_ and Z_2_ and 29% in Z_3_ and Z_4_) and Cu_400_ (41% in Z_1_ and Z_2_ and 28% in Z_3_ and Z_4_) compared to the control (Fig. 2A). The regions of Z_3_ and Z_4_ showed a better grain yield under high concentrations of Cu (Fig. 2A). The best zones for collecting *F. parviflora* seeds (tolerance to high concentrations of copper) for grain yield were identified as Z_3_ and Z_4_ zones.

In particular, exposure to Cu_50_ was better for *F. parviflora* to withstand the drought stress tensions. The reduction of grain yield at this concentration (Cu_50_) was less than other concentrations of copper (Cu_150_, Cu_300_, and Cu_400_) in response to drought stress (D_2_ and D_3_). At Cu_50_, the grain yield in D_2_ and D_3_ decreased (on average in four zones), respectively, 24% and 40% (Fig. 2B) compared to the control, while the reduction of this trait in moderate and severe drought (on average in four zones) was 28% and 45% in Cu_150_ and 43% and 55% in Cu_300_ and Cu_400_ (Fig. 2B) compared to the control. The highest value of grain yield in a combination of Cu and drought stresses was recorded at Cu_0_ and Cu_50_ under D_1_ treatment in all zones (55.87 g/m^2^). The lowest value was observed at Cu concentration of 400 mg/kg under D_3_ treatment in four zones (17.02 g/m^2^; Fig. 2B).

#### Malondialdehyde

**Figure 3:**
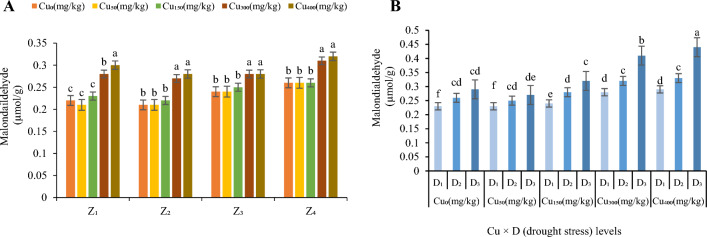
The effect of Cu stress on malondialdehide content of *F. parviflora* under field conditions (**A**). The means for the interaction effects of Cu and drought stresses on malondialdehyde (**B**). Different letters for each figure indicate significant differences at *p* < 0.05. Copper concentration (mg/kg). Seed originated zones Z_1_: Zone 1. Z_2_: Zone 2. Z_3_: Zone 3. Z_4_: Zone 4. D_1_: Control, D_2_: moderate drought stress; D_3_: severe drought stress.

Oxidative stress in term of high contents of MDA was observed in this study (Fig. 3). High concentrations of Cu in the soil significantly (*p* < 0.05) increased contents of MDA of *F. parviflora* leaves compared with control (Fig. 3A). MDA contents were increased averaged by 102% in Cu_50_, 104% in Cu_150_, 123% in Cu_300_ and 125% in Cu_400_ over all zones in the leaves (Fig. 3A). The maximum contents of MDA in leaves were in Cu_300_ and Cu_400_ (0.3 µmol/g in Z_1_, 0.28 µmol/g in Z_2_, 0.28 µmol/g in Z_3_ and 0.31 µmol/g in Z_4_; Fig. 3A) and the minimum contents were in Cu_0_ and Cu_50_ (0.21 µmol/g in Z_1_, 0.21 µmol/g in Z_2_, 0.24 µmol/g in Z_3_ and 0.26 µmol/g in Z_4_; Fig. 3A).

Elevated Cu led to significant increase in MDA content by 108% at Cu_0_, 114% at Cu_50_, 116% at Cu_150,_ 114% at Cu_300_ and 116% at Cu_400_ (average in four zone; Fig. 3B) at moderate drought and 117% at Cu_0_, 123% at Cu_50_, 133% at Cu_150,_ 146% at Cu_300_ and 152% at Cu_400_ at severe drought (average in four zone; Fig. 3B). Cu_400_ and severe drought showed the highest amount of MDA in all zones (0.47 µmol/g; Fig. 3B) and Cu_0_ and Cu_50_ under D_1_ treatment showed the lowest one (0.21 µmol/g; Fig. 3B).

#### Total soluble carbohydrates

**Figure 4:**
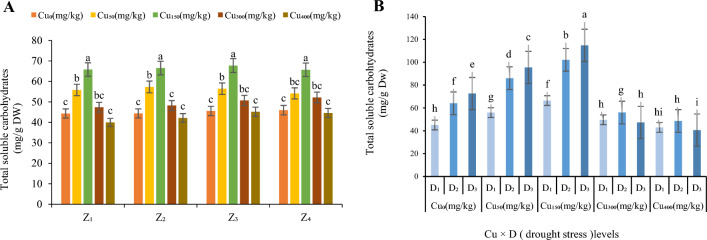
The effect of Cu stress on total soluble carbohydrates of *F. parviflora* under field conditions (**A**). The means for the interaction effects of Cu and drought stresses on total soluble carbohydrates (**B**). Different letters for each figure indicate significant differences at *p* < 0.05. Copper concentration (mg/kg). Seed originated zones Z_1_: Zone 1. Z_2_: Zone 2. Z_3_: Zone 3. Z_4_: Zone 4. D_1_: Control, D_2_: moderate drought stress; D_3_: severe drought stress.

Increasing Cu concentration up to Cu_300_ was associated with a significant (*p* < 0.05) increase in the contents of TSC (Fig. 4) of *F. parviflora* leaves. Compared to the control, the maximum TSC were in Cu_150_ at all zones (65.81 mg/g DW in Z_1_, 66.53 mg/g DW in Z_2_, 67.74 mg/g DW in Z_3_ and 65.69 mg/g DW in Z_4_; Fig. 4A) and the minimum was at Cu_400_ over all zones (39.95 mg/g DW in Z_1_, 42.16 mg/g DW in Z_2_, 45.17 mg/g DW in Z_3_ and 44.56 mg/g DW in Z_4_; Fig. 4A)_._ At 150 mg/kg of Cu, TSC increased by 151% compared to the control (Fig. 4A). Interaction of the stresses (Cu and drought) increased TSC content, by 142% at Cu_0_, 154% at Cu_50_, 155% at Cu_150_, 110% at Cu_300_ and 113% at Cu_400_ (average in four zone; Fig. 4B) in moderate drought and 161% at Cu_0_, 170% at Cu_50_, 172% at Cu_150_ and 96% at Cu_300_ and 90% at Cu_400_ (average in four zone; Fig. 4B) in severe drought stress. Cu_150_ under severe drought showed the highest amount of TSC in all zones (114.83 mg/g DW; Fig. 4B) and Cu 400 under severe drought showed the lowest one over four zones (40.62 mg/g DW; Fig. 4B).

#### Proline

**Figure 5:**
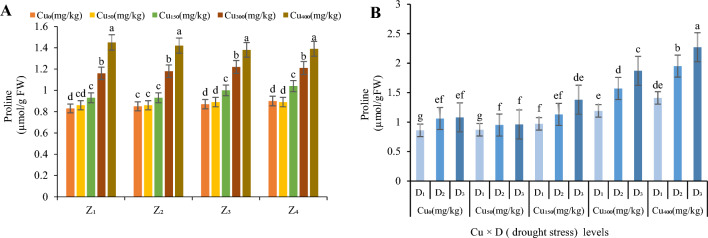
The effect of Cu stress on proline content of *F. parviflora* under field conditions (**A**). The means for the interaction effects of Cu and drought stresses on proline (**B**). Different letters for each figure indicate significant differences at *p* < 0.05. Copper concentration (mg/kg). Seed originated zones Z_1_: Zone 1. Z_2_: Zone 2. Z_3_: Zone 3. Z_4_: Zone 4. D_1_: Control, D_2_: moderate drought stress; D_3_: severe drought stress.

It was noticed that highest content of proline was observed under Cu_400_ concentration in all zones (1.45 µmol/g FW in Z_1_, 1.42 µmol/g FW in Z_2_, 1.38 µmol/g FW in Z_3_ and 1.39 µmol/g FW in Z_4_; Fig. 5A) and the least content of proline was in Cu_0_ in all zones (0.83 µmol/g FW in Z_1_, 0.85 µmol/g FW in Z_2_, 0.87 µmol/g FW in Z_3_ and 0.9 µmol/g FW gm^-2^ in Z_4_; Fig. 5A). Increasing Cu level till Cu_400_, significantly increased contents of proline in the leaves of *F. parviflora*. The averaged content of proline under Cu_300_ and Cu_400_ treatments was less (17%) than the averaged values for proline in Z_1_ and Z_2_ (Fig. 5A).

Both drought and Cu treatments had an increasing effect on proline content (Fig. 5B). Moderate drought increased proline content by 123% at Cu_0_, 106% at Cu_50_, 119% at Cu_150,_ 132% at Cu_300_ and 137% at Cu_400_ compared to the control (average in four zone (Fig. 5B). Severe drought also increased proline content by 126% at Cu_0_, 108% at Cu_50_, 145% at Cu_150_, 157% at Cu_300_ and 161% at Cu_400_ compared to the control (average in four zone; Fig. 5B). The highest value of proline content in a combination of Cu and drought stresses, recorded at Cu_400_ under D_3_ treatment in all zones (2.27 µmol/g FW; Fig. 5B) but the lowest one was observed at Cu_0_ under D_1_ treatment in four zones (0.86 µmol/g FW; Fig. 5B).

#### Anthocyanin

**Figure 6:**
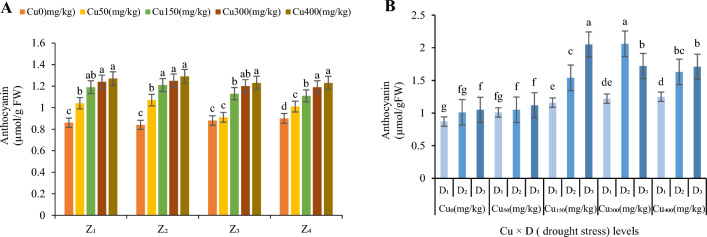
The effect of Cu stress on anthocyanin content of *F. parviflora* under field conditions (**A**). The means for the interaction effects of Cu and drought stresses on anthocyanin (**B**). Different letters for each figure indicate significant differences at *p* < 0.05. Copper concentration (mg/kg). Seed originated zones Z_1_: Zone 1. Z_2_: Zone 2. Z_3_: Zone 3. Z_4_: Zone 4. D_1_: Control, D_2_: moderate drought stress; D_3_: severe drought stress.

In the present study, increasing levels of Cu concentrations in soil up to Cu_400_, enhanced anthocyanin content in the leaves (Fig. 6A). Compared to the control, the highest content of the anthocyanin was in Cu_400_ over all zones (1.27 µmol/g FW in Z_1_, 1.29 µmol/g FW in Z_2_, 1.23 µmol/g FW in Z_3_ and 1.23 µmol/g FW in Z_4_; Fig. 6A) and the least one was in Cu_0_ (0.86 µmol/g FW in Z1, 0.84 µmol/g FW in Z2, 0.88 µmol/g FW in Z3 and 0.90 µmol/g FW in Z4; Fig. 6A)_._ Anthocyanin content, increased 117% at Cu_0_, 104% at Cu_50_, 134% at Cu_150_, 168% at Cu_300_ and 130% at Cu_400_ in moderate drought and 126% at Cu_0,_ 112% at Cu_50_, 174% at Cu_150_, 140% at Cu_300_ and 137% at Cu_400_ in severe drought (averaged in all zones; Fig. 6B). The highest value of anthocyanin content was recorded at Cu_300_ and moderate drought in all zones (2.06 µmol/g FW; Fig. 6B) and the minimum value was recorded at Cu_0_ without drought in all zones (0.87 µmol/g FW, Fig. 6B).

#### Total phenolics and total flavonoids contents

**Figure 7:**
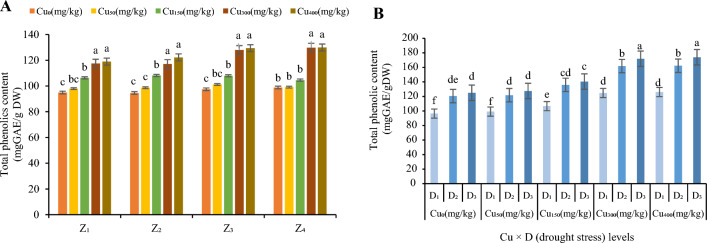
The effect of Cu stress on total phenolics content of *F. parviflora* under field conditions (**A**). The means for the interaction effects of Cu and drought stresses on total phenolics content (**B**). Different letters for each figure indicate significant differences at *p* < 0.05. Copper concentration (mg/kg). Seed originated zones Z_1_: Zone 1. Z_2_: Zone 2. Z_3_: Zone 3. Z_4_: Zone 4. D_1_: Control, D_2_: moderate drought stress; D_3_: severe drought stress.

**Figure 8:**
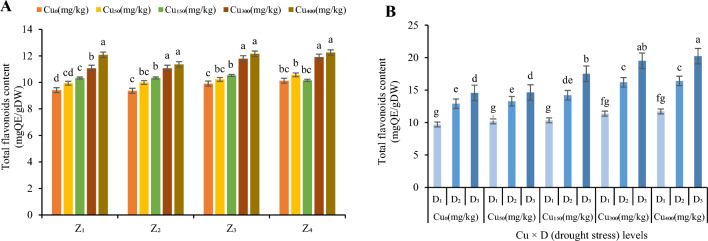
The effect of Cu stress on total flavonoids of *F. parviflora* under field conditions (**A**). The means for the interaction effects of Cu and drought stresses on total flavonoids (**B**). Different letters for each figure indicate significant differences at *p* < 0.05. Copper concentration (mg/kg). Seed originated zones Z_1_: Zone 1. Z_2_: Zone 2. Z_3_: Zone 3. Z_4_: Zone 4. D_1_: Control, D_2_: moderate drought stress; D_3_: severe drought stress.

In the present study, TPC and TFD were also measured from the leaves of *F. parviflora* under elevated levels of Cu in the soil (Figs. 7 and 8). These results suggested that TPC and TFD significantly (*p* < 0.01) increased as the Cu level in the soil rises compared with the control.

Contents of total phenolic and total flavonoids in Z_1_ and Z_2_ were 11.76% and 15.5% respectively more than Z_3_ and Z_4_ under high concentration of Cu (300 and 400 mg/kg; Figs. 7A and 8A). The minimum values were observed in the plants which grown without the Cu concentration in the soil (94.88 mg GAE/g DW in Z_1_, 94.57 mg GAE/g DW in Z_2_, 97.43 mg GAE/g DW in Z_3_ and 98.83 mg GAE/g DW in Z_4_ for TPC (Fig. 7A) and 9.42 mg QE/g DW in Z_1_, 9.37 mg QE/g DW in Z_2_, 9.91 mg QE/g DW in Z_3_ and 10.13 mg QE/g DW in Z_4_ for TFD (Fig. 8A). But these values increased continuously as the Cu level increases in the soil and the maximum contents of them were observed in Cu_400_ (129.95 mg GAE/g DW in Z_1_, 129.2 mg GAE/g DW in Z_2_, 122.35 mg GAE/g DW in Z_3_ and 122.92 mg GAE/g DW in Z_4_ for TPC (Fig. 7A) and 12.25 mg QE/g DW in Z_1_, 12.16 mg QE/g DW in Z_2_, 11.36 mg QE/g DW in Z_3_ and 11.08 mg QE/g DW in Z_4_ for TFD (Fig. 8A). In Cu_400_ the contents were increased by 137% for TPC and 127% for TFD (compared to the control).

The effects of two stresses (Cu and drought) increased TPC in all zones and this increase was significant (Fig. 7). Elevated Cu increased TPC at moderate drought stress by averaged 124% in Cu_0,_ 123% in Cu_50,_ 127% in Cu_150,_ 129% Cu_300_ and 129% in Cu_400_ over all zones (Fig. 7B) and in severe drought by averaged 131% in Cu_0,_ 129% in Cu_50,_ 131% in Cu_150,_ 139% in Cu_300_ and 140% in Cu_400_ over all zones (Fig. 7B). It was noticed that minimum values of TPC was recorded in a stress-free environment (Cu_0_ and D_1_; 96.42 mg GAE/g DW; Fig. 7B). The highest TPC was in Cu_400_ under severe drought over all zones (173.96 mg GAE/g DW; Fig. 7B).

The contents of TFD in the leaves of *F. parviflora* was increased with moderate drought by 119% at Cu_0_, 130% at Cu_50_, 137% at Cu_150,_ 152% at Cu_300_ and 148% at Cu_400_ and increased with severe drought by 150% at Cu_0_, 145% at Cu_50_, 169% at Cu_150_, 171% at Cu_300_ and 174% at Cu_400_ (Fig. 8B). The highest value of TFD in a combination of Cu and drought stresses in all zones was recorded at Cu_400_ under D_3_ treatment (20.23 mg QE/g DW_;_ Fig. 8B). The lowest TFD was observed at Cu concentration of 0 mg/kg under D_1_ treatment in four zones (9.7 mgQE/g DW; Fig. 8B).

#### Antioxidant activity

**Figure 9:**
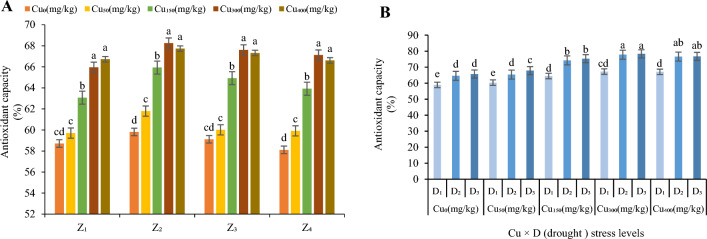
The effect of Cu stress on antioxidant activity of *F. parviflora* under field conditions (**A**). The means for the interaction effects of Cu and drought stresses on antioxidant activity (**B**). Different letters for each figure indicate significant differences at *p* < 0.05. Copper concentration (mg/kg). Seed originated zones Z_1_: Zone 1. Z_2_: Zone 2. Z_3_: Zone 3. Z_4_: Zone 4. D_1_: Control, D_2_: moderate drought stress; D_3_: severe drought stress.

Antioxidant activity of *F. parviflora leaves* were evaluated under various levels of Cu (0, 50, 150, 300 and 400 mg/kg; Fig. 9A). It was noticed that the antioxidant capacity increased when plants were subjected to high concentration of copper (400 and 500 mg/kg). Our results depicted that antioxidant capacity increased in the leaves by 102% in Cu_50_, 109% in Cu_150_, 114% in Cu_300_ and 113% in Cu_400_ compared to the control. The maximum antioxidant capacity was observed in the plant grown under Cu level of 300 and 400 mg/kg (66.72% in Z_1_, 68.26% in Z_2_, 67.61% in Z_3_ and 67.12% in Z_4_; Fig. 9A) and the minimum antioxidant capacity was observed in a stress-free environment (58.71% in Z_1_, 59.81% in Z_2_, 59.11% in Z_3_ and 58.11% in Z_4_; Fig. 9A). Antioxidant capacity was increased by interaction of drought and Cu hence the interaction between the two stresses was significant (Fig. 9B). Antioxidant capacity was increased by 110% in Cu_0,_ 108% in Cu_50,_ 115% in Cu_150,_ 116% in Cu_300_ and 115% in Cu_400_ in moderate drought and 110% at Cu_0,_ 112% in Cu_50,_ 117% in Cu_150,_ 118% in Cu_300_ and 116% in Cu_400_ in severe drought (average in four zone, Fig. 9B). The lowest value of antioxidant capacity was recorded at Cu_0_ and D_1_ treatment (58.93%; Fig. 9B) while the highest one was recorded at Cu_300_ and severe drought (78.34%; Fig. 9B).

In a total result, averaged over four studied zones, the highest values for different studied traits (averaged over four studied zones) was observed in this order: grain yield (56.64 g/m^2^) under Cu_50_ × D_1,_ anthocyanin (2.14 µmol/g FW) under Cu_300_ × D_2,_ TSC (115.23 mg/g DW) under Cu_150_ × D_3_ and DPPH (83.95%) under Cu_300_ × D_3._ The highest values for MDA (0.47 µmol/g), proline (2.45 µmol/g FW), TPC (188.99 mg GAE/g DW) and TFD (22.1 mg QE/g DW was observed at Cu_400_ × D_3._ The least value for MDA (0.21 µmol/g), proline (0.83 µmol/g FW), anthocyanin (0.87 µmol/g FW), TPC (94.88 GAE/g DW), TFD (9.42 mg QE/g DW) and DPPH (58.71%) was observed under non-stress condition (Cu_0_ × D_1_)_,_ but the least values for grain yield (14.42 g/m^2^) and TSC (36.48 mg/g DW) was observed under the interaction Cu_400_ × D_3_ treatment.

### The effects of Cu stress on studied traits under non-stress and drought stress

#### Heat map analysis

**Figure 10:**
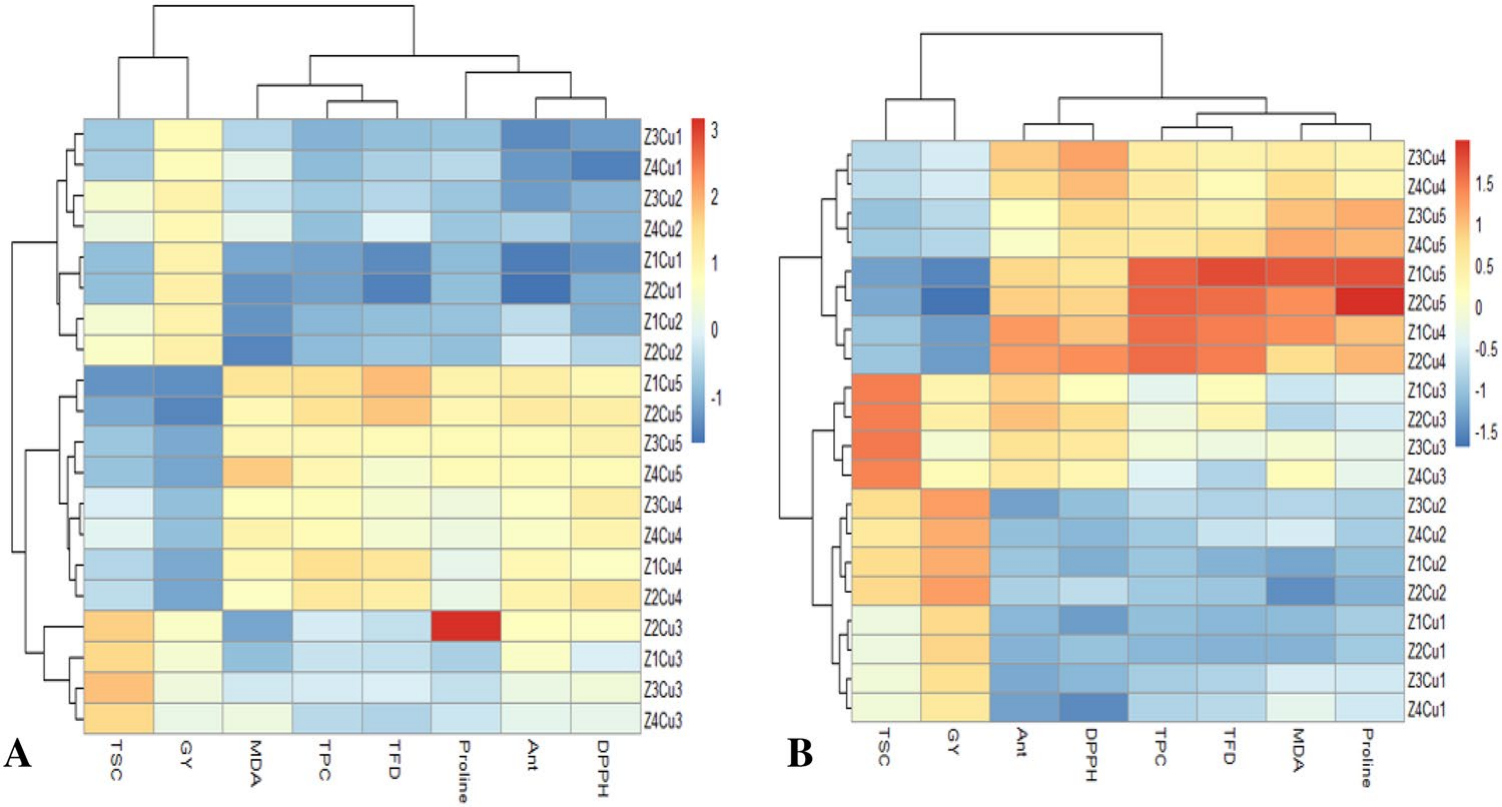
Heat Map graph showing the clustering of 20 environments (Z × Cu) based on their studied traits under non- drought (**A**) and drought- stress (**B**) conditions. *TPC* total phenolics content, *TFD* total flavonoids, *Ant* anthocyanin, *MDA* malondialdehyde, *TSC* total soluble carbohydrates, *DPPH* 2,2-diphenyl-1-picrylhydrazyl, *GY* grain yield.

The Heat Mam graph was drawn to gain a better understanding of the treatments clustering (Cu × Z) based on their different studied traits in two different non- drought (A) and drought-stress (B) environments (Fig. 10). Clearly, the treatments (Cu × Z) may be can be categorized into 7 different ones, which according map legend, seven different color represented different ranges for traits values from Z_1_Cu_1_ to Z_4_Cu_5_, demonstrating the relative value of each under these 20 different treatments. As presented in heat map graphs (Fig. 10 A and B), the synergism effects of drought stress were significantly obvious on different biochemical traits under Cu stress. The comparison between two condition including non- drought (Fig. 10A) and drought- stress (Fig. 10B) implied at significant increase in MDA, DPPH, TPC, TFD, and anthocyanin content, specially under Cu_300_ and Cu _400_ (mg/kg) drought stress (Fig. 10B) Vice versa, a sharp decrease observed in grain yield under higher mentioned Cu concentrations (Fig. 10B).

#### Principle component analysis

**Figure 11:**
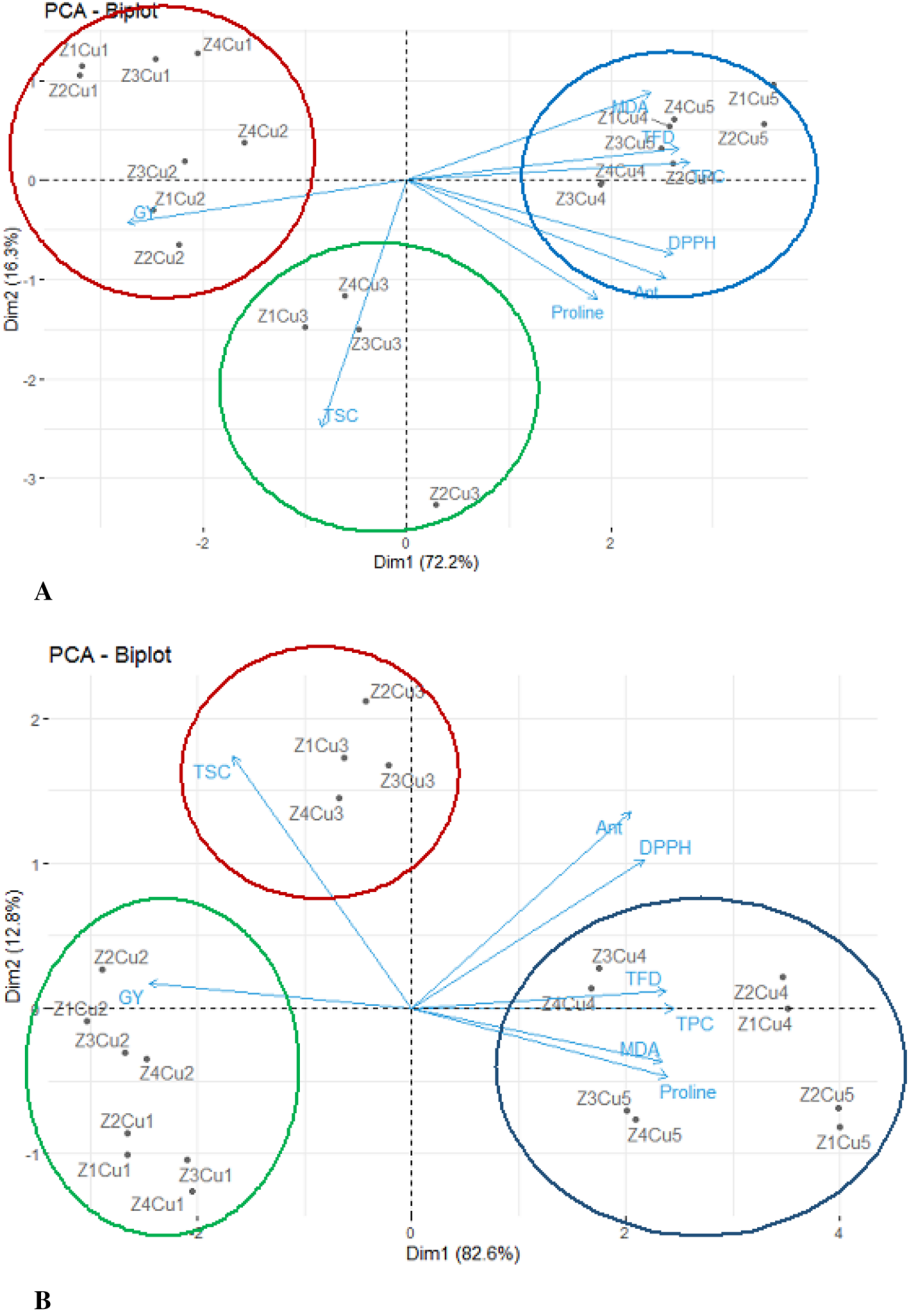
Traits- environment biplot for *F. parviflora* under the non- drought stress (**A**) and drought- stress (**B**) averaged over two mineral regions. *TPC* total phenolics content, *TFD* total flavonoids, *Ant* anthocyanin, *MDA* malondialdehyde, *TSC* total soluble carbohydrates, *DPPH* 2,2-diphenyl-1-picrylhydrazyl, *GY* grain yield.

A biplot, as a result of Principle Component Analysis (PCA) done as an efficient multivariate tool for the interpretation of data, was constructed based on the first (PC_1_) and (PC_2_) principal components for different studied traits under the interaction of Cu × Z (Zone) treatments under non-drought and drought–stress conditions (Fig. 11A,B). Based on the studied traits represented in the biplot, the first (PC_1_) and second (PC_2_) components explained about 72.2% and 16.3% of the total variance of the traits under non- drought condition, respectively (Fig. 11A); thus, both PCs cumulatively described vc 88.5% of the total variance of all the traits analyzed. The highest values for TFD, TPC, DPPH, anthocyanin and proline was identified under the Cu × Z treatments in blue color croup (Fig. 11A). On the other hand, the treatment in red group had the highest positive values for grain yield (Fig. 11A). Finally, the Cu × Z treatments in green color had the highest values for TSC (Fig. 11A). Under water- deficit condition, in 82.6% and 12.8% of the total variance was explained by PC_1_and PC_2_, respectively (Fig. 11B). Therefore, both PCs explained 95.4% of the total variance in all the traits investigated. Below drought stress, the plant under the Cu × Z treatments in blue color group, had the highest TFD, TPC, MDA and proline content (Fig. 11B). The plants under Cu × Z treatments in red color group had the highest TSC (Fig. 11B). Finally, the highest grain yield was observed under Cu × Z treatments in green color (Fig. 11B).

## Discussion

Abiotic stresses are major constraints for plants growth worldwide^27,28^ and exacerbate yield loss under changing climatic conditions^28^. Heavy metals and drought are generally considered important stresses in plant production systems^29^. Copper, as micronutrient is essentially required for redox-active transition and as a cofactor for several enzymes which involved in many biochemical processes including photosynthesis, respiration and cell wall metabolism^30,31^. It has been acknowledged that Cu plays an important role in drought tolerance in different plant species through promoting the photosynthesis pigments and contribute to organic matter accumulation under water deficit conditions^30,32^. The soil pollution with heavy metals has become a serious environmental problem around the world due to industrialization and soil pollution^26^, specially under arid and semi-arid regions. Metal-rich soils often have low organic matter content, resulting in low water-holding capacity and subsequent reduction in hydraulic and stomatal conductance^33^. Therefore, the collection of these responses may aggravate the drought stress^24^. In this respect, substantial efforts have widely been made in previous years to identify the independent effects of heavy metals and drought stress on plants, but little attention has been paid to the interactive effects of these two stresses on plants. Here, the single and combined effects of Cu and drought stress were evaluated on different biochemical traits of *F. parviflora* which their seeds were collected from four different zones (Z_1_, Z_2_, Z_3_ and Z_4_) from two copper mines. Regarding the effects of different zones (Z_1_, Z_2_, Z_3_ and Z_4_) on grain yield and biochemical traits of the plants grown in the field, it should be acknowledged that under both drought and non-drought conditions, the originated plants from Z_3_ and Z_4_ regions showed lower levels of grain yield and higher levels of physiological traits than Z_1_ and Z_2_ under high concentrations of Cu (Cu_300_ and Cu_400_). This finding can be resulted from the higher genetic and/or edaphic acclimatization of *F. parviflora* seeds originated from Z_3_ and Z_4_ regions to combat the Cu toxicity stress. It the other hand, Z_3_ and Z_4_ were identified as the best areas for collecting *F. parviflora* seeds and cultivation on contaminated soils with Cu to achieve expected grain yield.

In the present study, the effects of Cu stress showed adverse effects on grain yield of the *F. parviflora*. The application of Cu_0_ and Cu_50_ were suitable for grain yield, but with increasing Cu concentrations, the grain yield significantly (*p* < 0.01) was decreased. Under Cu_50_ × drought stress interactions, Cu showed adverse effect on drought stress compared to the control. Here, low concentrations of Cu (50 and 150 mg/kg) were able to improve grain yield loss under different levels of drought stress (D_2_ and D_3_). It could be concluded that under low Cu concentrations, it acts as a micronutrient, with triggering effects to increase plant tolerance to drought stress through the increase in water holding capacity and biomass accumulation^30,32^. This finding was similar to positive effects of Zn and Fe microelements on increase of grain yield in *Thymus vulgaris*^34^ and *Ocimum basilicum*^35^. Combined effects of severe drought and high concentrations of Cu (300 and 400 mg/kg) may affect nutrients uptake and allocation within fumitory tissues due to disruption of water homeostasis, ionic allocation and cell permeability barriers, which led to a significant decrease in grain yield^12^. Also, under such condition, defense mechanisms could only sustain plant until the end of the growing season and grain yield at these levels of stress severely reduced. High concentrations of heavy metals reduce the synthesis of chlorophyll, photosynthetic activities, inhibiting activities of the Calvin cycle and the activity of enzymes related to soluble carbohydrates production, which causes a decrease in grain yield in areas with high concentrations of Cu^36,37^.

Malondialdehyde (MDA) often used as a marker for measure of oxidative stress in cell membranes^38^. Under different oxidative stresses, membrane peroxidation led to an increase in the content of lipid peroxidation which produces MDA in the tissues^38^. Here, Cu toxicity may exert in electron transport and respiration like subcellular organelle functions, which generate hydroxyl radical due to the decomposition of H_2_O_2_ and subsequent increase in lipid peroxidation of cell membranes^39^. It was observed that *F. parviflora* had no significant increase in MDA content under interactions of lower Cu (Cu_50_ and Cu_150_) and drought stresses (D_2_ and D_3_), but the plants under drought and high Cu concentrations (Cu_300_ and Cu_400_) showed higher MDA content rather than their content under single stresses of drought (D_1_ and D_2_) and or Cu (50, 150, 300 and 400 mg/kg), which demonstrate a synergism effects of Cu and drought stress on lipid peroxidation of membranes rather than singular stresses.

Plant adaptation to oxidative stress is associated with different metabolic adjustments such as accumulation of soluble carbohydrates^40^, which reveal two defensive mechanisms including osmotic adjustment and cellular compatibility^41^. According to the results, increasing Cu concentration up to 150 mg/kg under moderate and severe drought stress showed modulatory roles on increase in TSC under drought stress conditions, however, exposure to higher concentration (Cu_300_ and Cu_400_) aggravates drought stress in an additive manner, which led to decrease in the production of TSC, making the plants more vulnerable to drought stress. This phenomenon may be linked to this fact that under low-medium concentrations of heavy metals like Cu, plants produce osmolytes such as TSC^14,42^, but at higher concentration (Cu_400_) increase generation of toxic free radicals which induce oxidative stress and subsequent inhibition in the production of TSC^43^. This could be due to the reduction of CO_2_ fixation in heavy metals treated plants at high concentrations, but in lower concentrations, the photosynthetic measurements were not affected following exposure, and subsequently cause a general increase in carbohydrate content^44^. Similarly, the decrease in TSC was previously reported under the high concentrations of Cu stress in *Trigonella foenum-graecum*^39^ and *Kandelia obovate*^45^. In concentration of Cu_150_, the content of TSC was significantly higher than single stresses of drought and Cu and control treatment (Cu_0_ D_1_), which led to transfer of more carbohydrates to grains and produce greater seed yield under combined stresses rather than single and/or control conditions. All morpho-physiological and biochemical changes under stress conditions are occurred for better compatibility of *F. parviflora* to employed stresses, which are associated with synthesis of TSC compounds.

Proline as an important compatible osmolyte in plants, plays multifarious roles in plant compromising adaptation, recovery, and signaling when it comes to combating stress^46^. Higher accumulation of proline, protects plants with maintaining cell turgor under stress conditions. Under drought stresses the occurrence of water imbalance leads to increase in the content of proline through osmotic regulation function^14,46,47^, but under heavy metals stress, proline increase the tolerance of plants through detoxifying the heavy metal effects in the cytoplasm and regulating the intracellular redox homeostasis potential by osmotic adjustments^48^.

In the present study, a significant (*p* < 0.01) increase was occurred in proline content with increasing the levels of Cu concentration, similar to the increase in proline content under Cu stress for *Cinnamomum camphora*^27^ and *Astragalus tragacantha*^49^. Here, the proline content of *F. parviflora* showed a significant increase after exposure to Cu treatment, because the applied concentration of Cu (up to 400 mg/kg) is under the tolerance threshold of fumitory ecotype. The secondary effects of drought (D_1_ and D_2_) and Cu (up to 400 mg/kg) stresses led to more accumulation of proline compared to the control.

Anthocyanins as a subclass of flavonoids^50^ have an important role in inducing and/or modulating array of different environmental agents including heavy metals^1,51^ for ROS quenching^52^, photo-protection and stress signaling^53^. Here, single effects of Cu (50 to 400 mg/kg) stress led to increase in anthocyanins content, which was similar to the other heavy metals application such as arsenic in *Lemna gibba* L.^54^ Cu, Zn, Mn, Pb and Hg in *Arabidopsis*^55^, Cu and Zn in *Capsicum annum* L.^56^, and in contrast with the report in annual Halophytes^51^. The chemical reactivity of anthocyanins depends on their capacity to metal chelation^51^. The most common metals that can form complexes with anthocyanins are Cu, Fe, Mg, Sn and K^57^. The combined effect of drought stress and Cu has not been previously reported on anthocyanin content in medicinal plant species. Under the combination of all drought stress levels with Cu_50_ (D_2_ × Cu_50_ and D_3_ × Cu_50_) the anthocyanin content was more than that of single effects of drought stress (D_1_ × Cu_50_), showing that Cu could mitigate the negative effects of drought in plants (Waraich et al., 2011). Under interaction of higher concentrations of Cu (Cu_150_, Cu_300_, and Cu_400_) and drought stress (D_2_ and D_3_), the dose-dependent trend was observed on the changes of anthocyanin. Hovered, under moderate and severe drought conditions, the highest content of anthocyanin was observed at 300 mg/kg (2.1 µmol/g FW) and 150 mg/kg (2.1 µmol/g FW) of Cu application, respectively. Although the greater degradation of chlorophyll molecules caused an increase in protective pigments of anthocyanin under drought stress^58^, the suppressing effects of high concentration of Cu (300 and 400 mg/kg) on anthocyanin content was observed under drought stresses treatments (D_2_ and D_3_).

Plants increase the contents of two major groups of SMs (phenolics and flavonoids) as effective antioxidants to avoid the oxidative damage which inhibit the over-generation of ROS under heavy metal stresses^52,59,60^ or in combination with other environmental stresses^12^. Phenoilcs and flavonoids biosynthesis pathways provide compounds involved in defense responses against a certain type of stresses such as heavy metals^52,61^. Phenolic compounds, could be also substrates for different peroxidases, which are the first line of defense against Cu toxicity^37^. Under heavy metal stresses, flavonoids enhance the process of metal chelation, which may reduce the level of harmful hydroxyl radicals in plant cells^62^. In the present study, increasing drought stress intensity led to an increase in TPC and TFD, which were consistent with the previous reports in *Carthamus tinctorius*^63^ and *Eruca sativa*^64^. Also, the increase in Cu concentrations up to Cu_400_, resulted in an increase in TPC and TFD. These findings were similar to the previous reports on tomato^65^, *Mentha spicata*^12^, wheat^61^, *Withania somnifera* L.^66^, *Gymnema sylvestre*^67^ and *Erica andevalensis*^68^. In contrary, a dose-dependent decrease was reported in TPC under the heavy metal stresses (Pb and Cu) in *Citrus aurantium* L. and cadmium in *Prosopis glandulosa*^69^. These discrepancies trends could be attributed to the differences in heavy metal type and its intensity, plant developmental stages and differences in plant species^63^. Here, the TPC and TFD of the *F. parviflora* showed upward trend under different interaction of Cu and drought stress compared to the single effects of drought and Cu levels. Therefore, incidence of combined stresses of Cu and drought have not necessarily adverse effects on SMs metabolism, because *F. parviflora* plants were able to activate the production of SMs to defend oxidative stress damage via ROS scavenging.

The effects of moderate drought stress on antioxidant activity under all levels of Cu treatment were not statistically significant compared to the control, but under severe drought (D_3_), the increase in antioxidant activity (DPPH%) of the *F. parviflora* leaves was higher under the both drought and Cu stresses than single treatments of Cu and/or drought, which demonstrated at synergism effects of severe drought and higher Cu concentrations on increasing the antioxidant activity. Conclusively, the findings of this study showed that Cu (at 50 mg/kg) as a micronutrient reduced the deleterious effects of drought stress in *F. parviflora*. Under interaction of D × Cu (150 mg/kg), and D × Cu (300 mg/kg), *F. parviflora* could withstand the stress by using some defense mechanisms such as increase in the contents of total soluble carbohydrates and anthocyanins, respectively. Under higher concentrations (Cu_300_ and Cu_400_), Cu addition could increase tolerance of *F. parviflora* to the osmotic stress caused by drought mainly by increasing the contents of total phenolics and total flavonoids. Furthermore, the ascending trend in the values of total phenolics and flavonoids was observed (from Cu_0_ to Cu_400_), which demonstrated at high threshold and pre-dominant role of non-enzymatic antioxidants in fumitory tolerance under combined stresses of Cu (up to 500 mg/kg) and drought.

## Conclusions

The results from this research could help our understanding of how medicinal plants act to polluted regions with Cu as a heavy metal under drought conditions. The findings supported the use of *F. parviflora* as a suitable species for contaminated soils with low and moderate Cu (50–150 mg/kg) under moderate drought stress. Based on these findings, it can be concluded that *F. parviflora* can cope with Cu stress due to active antioxidant defense system. The results could provide a criterion for the selection of tolerance species for better acclimatization under Cu mines and/or agricultural contaminated soils. More researches are required to evaluate the phyto-extraction of Cu in plants grown in the contaminated soils of arid regions.

## Materials and methods

### Plant materials and treatments

Two mineral regions (R_1_: Askary and R_2_: Rabor) including copper were studied from Kerman, Iran (Fig. S1), which their climatic and soil characteristics is presented in Table 2. Each mine (R_1_ and R_2_) were divided into four zones based on the Cu concentration in these four zones (Z_1_:50 mg/kg, Z_2_: 150 mg/kg, Z_3_: 300 mg/kg and Z_4_: 400 mg/kg). Then, seeds of the *F. parviflora* were collected from the growing plants four different zones for the aim of field experiment. The experimental design was conducted as a split factorial experiment based on a randomized complete block design (RCBD) design (replication = 3) was carried out at the research field of Payam -E- Noor University of Kerman (longitude 57° 06ʹ 21.22ʺ and latitude 30° 15ʹ 32.89ʺ, 1766 m ASL), Iran in 2019. Different levels of drought stresses (D_1_: non-stress, D_2_: moderate stress and D_3_: severe stress) were considered as main plots. Different Cu concentrations (control, 50, 150, 300 and 400 mg/kg) and four different zones (Z_1_, Z_2_, Z_3_, Z_4_) were considered as sub factors. Three number of rows were planted in each plot (2.5 m × 2 m). The distance between and within rows were 50 cm and 15 cm, respectively. Plants were irrigated uniformly under different conditions (D_1_, D_2_ and D_3_) until the 6–8 leaves stage and after that time drought and Cu stresses were established.

**Table 2:** Meteorological and soil characteristics of two mineral regions (R1 and R2) located in Kerman, Iran.

Region	Longitude	Latitude	Average height(m)	Average precipitation (mm)	Average temperature (°c)	Cu concentration (mg/kg)			
						Z_1_	Z_2_	Z_3_	Z_4_
R1	56° 54ʹ 30.89ʺ	31° 16ʹ 18.94ʺ	2111	219	17	50	150	300	400
R2	56° 17ʹ 59ʺ	32° 51ʹ 33ʺ	2176	321	15	50	150	300	400
Soil texture		Soil characteristics							
		pH	EC (dsm^–1)^	OM (%)	CaCO_3_ (%)				
R1	Loamy sandy	7.6	1.5	0.6	5.1				
R2	Loamy sandy	6.9	1.73	0.5	6.2				

R_1_: Askari region, R_2_: Rabor region. Z_1_: Zone 1, Z_2_: Zone 2, Z_3_: Zone 3, Z_4_: Zone 4.

The irrigation time was estimated according to cumulative evaporation (CE) (mm) from CLASS A pan. Under D_1_ treatment, the irrigation was done when cumulative evaporation (CE) reached to 75 (± 4) from evaporation pan (Class A). Under D_2_ treatment, the irrigation was done after 110 (± 4) mm CE. Under D_3_ treatment, the irrigation was done after 135 (± 4) mm CE. A pumping station via polyethylene pipes and a volumetric counter were used for irrigation system. The soil available water (SAW) is equivalent to difference between the volumetric water content at the permanent wilting point (${\theta }_{PWP}$) and at field capacity (${\theta }_{FC}$). In this study, volumetric soil water content at FC (– 0.03 MPa) and PWP (– 1.5 MPa) were 25% and 15%, respectively. The levels of irrigation treatments including D1, D2 and D3 were equivalent to 50%, 70% and 85% of depletion from SAW. The amount of irrigation water applied calculated as $I=\left[\frac{\left({\theta }_{FC}-{\theta }_{PWP}\right)Z\cdot {\rho }_{b}}{100}\right]$, which, I is irrigation depth (cm), θ_FC_ is soil gravimetric moisture percentage at field capacity (%), θ_PWP_ is soil gravimetric moisture percentage at irrigation time (20%), Z is root zone depth (cm) (0.3 m), and ${\rho }_{b}$ is soil bulk density in the root zone (1.4 g cm^–3^)^70^. For applying Cu stress, final concentrations of Cu (0, 50, 150, 300, and 400 mg kg^–1^) were prepared by dissolving different amounts of CuSO_4_. 5H_2_O (Merck. Com., Germany) in a specific amount of distilled water and then sprayed on the soil surface. Then, the soil of each plot was mixed uniformly. Different biochemical traits were measured at full maturity stage based on three replications for each treatment.

### Malondialdehyde determination

For estimation malondialdehyde (MDA) content, 3 mL of 0.5% TBA and 2 mL of extraction solution were aggressively mixed together for this experiment^63^. The mixture was heated for 30 min at 95 °C in a water bath of constant temperature before cooling to room temperature with ice. The supernatant was discovered at wavelengths of 450, 532, and 600 nm after 15 min of centrifugation at 5000×*g*.

### Proline determination

The content of proline was measured according to Bates et al.^71^. The fresh leaf samples (0.2 g) were first given 3 ml of sulphosalicylic acid (3% w/v). The mixture was then centrifuged for 15 min at 18,000×*g*. Glacial acetic (CH_3_COOH) (2ml) and the ninhydrin (C_9_H_6_O_4_) (2ml) reagent were added to the test tubes after the supernatant (2 ml) was transferred to a fresh tube. The reaction mixture was heated for 60 min in a water bath before cooling on ice. Then 4 ml of toluene was then added, and it was left at room temperature for 30 min. After the separation of upper phase by shaking the tubes for 15 s, a spectrophotometer (Unico- UV 2100, USA (was used to detect its absorbance at 520 nm.

### Total soluble carbohydrates assay

A test tube containing 2 mL of a carbohydrate solution and 1 mL of a 5% aqueous phenol solution is used to measure the total soluble carbohydrates (TSC). The liquid is then quickly added to 5 mL of pure sulfuric acid. The test tubes are vortexed for 30 s after standing for 10 min, and then they are placed in a water bath at room temperature for 20 min to allow for color development. After that, a spectrophotometer records the light absorption at 490 nm^72^.

### Anthocyanins determination

Fresh leaf tissues (0.1 g) were homogenized in 2 mL of acidified methanol (1% HCI) in the initial stage. This was done at room temperature^73^. The entire extract was centrifuged for 25 min at 4000 rpm after one day. Then, using a spectrophotometer and a wave length of 511 nm, the anthocyanin (Ant) content was calculated using the extinction coefficient of raphanusin (33,000 M^−1^ cm^−1^).

### Total phenolics content determination

The Folin-Ciocalteu technique was used to estimate the total phenolics content (TPC)^74^. Briefly, 2.3 mL of deionized water was added to a 15-mL tube before 0.1 mL of the extract sample was added. Folin-Ciocalteu reagent (1:1) was added to the mixture in a volume of 0.4 mL. Then, 1.2 mL of 20% sodium carbonate was then added after the mixture had been allowed to stand at room temperature for 7 min. For 60 min, the response was permitted to proceed. A Unico- UV 2100 (USA) spectrophotometer calibrated with gallic acid (GA) was used to measure absorbance at 765 nm against a blank.

### Total flavonoids determination

The colorimetric AlCl_3_ method was used to determine each fraction's total flavonoid (TFD) concentration^75^. Separately, 1.5 mL of methanol, 0.1 mL of 10% aluminum chloride, 0.1 mL of 1 M potassium acetate, and 2.8 mL of deionized water were combined with 0.5 mL of the properly diluted sample solutions. This mixture was then left at room temperature for 30 min. A Unico- UV 2100 (USA) spectrophotometer was used to test the reaction mixture's absorbance at 415 nm. Quercetin equivalents (QE) per gram of dry extract were used to measure the results.

### Determination of free radical scavenging activity

The DPPH (2,2-Diphenyl-1-picrylhydrazyl) radical-scavenging activity test procedure was carried out to estimate the antioxidant activity^63^. At 515 nm, a DPPH (Sigma-Aldrich) absorbance reduction was observed. Ascorbic acid (Sigma-Aldrich) served as the positive control. The equation: inhibition concentration (inhibition percentage: IP_%_) = (absorbance control-absorbance sample)/(absorbance control) × 100 was used to quantify the DPPH radical scavenging activity.

### Statistical analysis

The two- way analysis of variance (ANOVA) was performed using SAS software (ver. 9.1) to determine the significant differences among various treatments. The traits mean comparison carried out based on the Least Significant Difference (LSD) test (*P* < 0.05). All data were presented as trait mean ± standard error (SE). The biplot for Principal Component Analysis (PCA) was performed using the R software (Ver. 3.6.1). The Heatmap graphs were performed using “pheatmap” package ver. 1.0. 12^76^.

### Ethical standards

This research study was carried out in accordance with the national, international or institutional guidelines.

### Plant guideline statement

All the experiments and collection of plant materials were performed in accordance with relevant guidelines and regulations.

### Supplementary Information

Supplementary Figure S1.

## Data Availability

The raw data of this article will be made available by corresponding author (Dr. Pooran Golkar; golkar@iut.ac.ir), according to the personal requests.
