# Publisher Correction: Physiological and biochemical mechanisms of grain yield loss in fumitory (*Fumaria parviflora* Lam.) exposed to copper and drought stress

**DOI:** 10.1038/s41598-023-46587-x

**Published:** 2023-11-07

**Authors:** Mansoureh Tashakorizadeh, Pooran Golkar, Mohammad Reza Vahabi, Mansour Ghorbanpour

**Affiliations:** 1 Forests and Rangelands Research Department, Kerman Agricultural and Natural Resources Research and Education Center, Agricultural Research Education and Extension Organization (AREEO), Kerman, Iran; 2 Department of Natural Resources, Isfahan University of Technology, Isfahan, 8415683111 Iran; 3 Department of Medicinal Plants, Faculty of Agriculture and Natural Resources, Arak University, Arak, 38156-8-8349 Iran

Correction to: *Scientific Reports* 10.1038/s41598-023-45103-5, published online 20 October 2023

In the original version of this Article, the title of Table 2 was omitted. The correct Table 2 appears below.

**Table 2:** Meteorological and soil characteristics of two mineral regions (R_1_ and R_2_) located in Kerman, Iran.

Region	Longitude	Latitude	Average height(m)	Average precipitation (mm)	Average temperature (°c)	Cu concentration (mg/kg)			
						Z_1_	Z_2_	Z_3_	Z_4_
R1	56° 54ʹ 30.89ʺ	31° 16ʹ 18.94ʺ	2111	219	17	50	150	300	400
R2	56° 17ʹ 59ʺ	32° 51ʹ 33ʺ	2176	321	15	50	150	300	400
Soil texture		Soil characteristics							
		pH	EC (dsm^–1)^	OM (%)	CaCO_3_ (%)				
R1	Loamy sandy	7.6	1.5	0.6	5.1				
R2	Loamy sandy	6.9	1.73	0.5	6.2				

R_1_: Askari region, R_2_: Rabor region. Z_1_: Zone 1, Z_2_: Zone 2, Z_3_: Zone 3, Z_4_: Zone 4.

The original Article has been corrected.

