# Supplementary material for: Physiological and biochemical mechanisms of grain yield loss in fumitory (Fumaria parviflora Lam.) exposed to copper and drought stress
Source: Sci Rep. 2023 Oct 20;13:17934. doi: 10.1038/s41598-023-45103-5 (PMC10589251; doi:10.1038/s41598-023-45103-5)
Supplement: Supplementary file 1 — Supplementary Figure S1. [file 41598_2023_45103_MOESM1_ESM.docx]

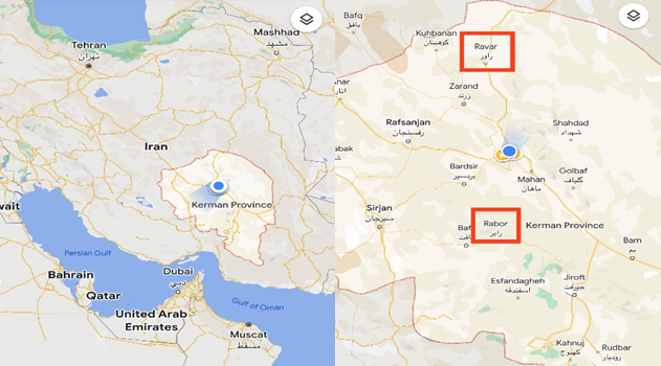


Fig S1. The geographical location of two copper mines (Rabor and Askary) in Kerman province, Iran 4 (https://maps.google.com).
